# Rituximab for Autoimmune Encephalitis with Epilepsy

**DOI:** 10.1155/2020/5843089

**Published:** 2020-06-23

**Authors:** Mohankumar Kurukumbi, Rahul H. Dave, Jose Castillo, Tulsi Shah, Joanne Lau

**Affiliations:** ^1^Department of Neurology, Inova Fairfax Hospital, Falls Church, VA, USA; ^2^Virginia Commonwealth University School of Medicine, Inova Campus, Falls Church, VA, USA

## Abstract

Intractable epilepsy remains a significant medical challenge, resulting in recurrent and prolonged intensive care unit (ICU) admissions. Autoimmune encephalitis is emerging as a treatable cause of intractable epilepsy. It is characterized by antibodies against cerebral antigens, such as potassium channels such as leucine-rich, glioma inactivated 1 (LGI1) and contactin-associated protein 2 (CASPR2), calcium channels such as the voltage-gated calcium channel (VGCC), or neurotransmitter receptors such as the *α*-amino-3-hydroxy-5-methyl-4-isoxazolepropionic acid receptor (AMPAR), gamma aminobutyric acid receptor (GABAR), and *N*-methyl-D-aspartate receptor (NMDAR). Diagnosis requires a syndrome consistent with an antibody identified in serum or cerebrospinal fluid (CSF) using methods that minimize risk of false-positives. Although there is no officially approved therapy for these disorders, typical approaches involve chronic high-dose steroids, intravenous immunoglobulin (IVIG), or plasma exchange. Rituximab is effective for antibody-associated disorders such as lupus, myasthenia gravis, and neuromyelitis optica. Here, we present three patients who were admitted with recalcitrant status epilepticus and demonstrated serum antibodies against NMDAR, LGI1, or VGCC using a cell-based assay. All patients demonstrated complete, long-term epilepsy control and improvement in symptoms with rituximab.

## 1. Introduction

Autoimmune encephalitis is now well recognized as a treatable cause of intractable epilepsy. Certain antibodies have been identified as likely culprits of these syndromes, and early diagnosis with prompt treatment is the mainstay understanding when combating these conditions. Therapy for these disorders include largely some form of immunosuppressants, with steroids, IVIG, or plasma exchange in some combination being the commonly used first line options. Rituximab has been shown to be a promising medication to administer in other autoimmune disorders when the first line immunosuppressive agents fail to control the condition, but its use for autoimmune encephalitis remains unconfirmed. In this case series, we will be presenting three cases that presented with epilepsy and were all found to subsequently have the respective antibodies known to be associated with a specific autoimmune encephalitis, and we demonstrated symptom control with the use of rituximab.

## 2. Case Presentations

### 2.1. Case 1

A 32-year-old female with no history of seizures presented with an acute onset of behavioral changes and witnessed seizure activity. Behavioral changes included uncontrolled laughter, screaming, signs of agitation, spitting on the floor, complete lack of appetite, and foreign accent syndrome. She had two seizures characterized by tongue biting with postictal confusion and agitation. Initial head computed tomography (CT), urine toxicology, and serum electrolytes were normal. She was discharged on 500 mg of levetiracetam by mouth twice a day and plans for magnetic resonance imaging (MRI) as an outpatient.

**Figure 1:**
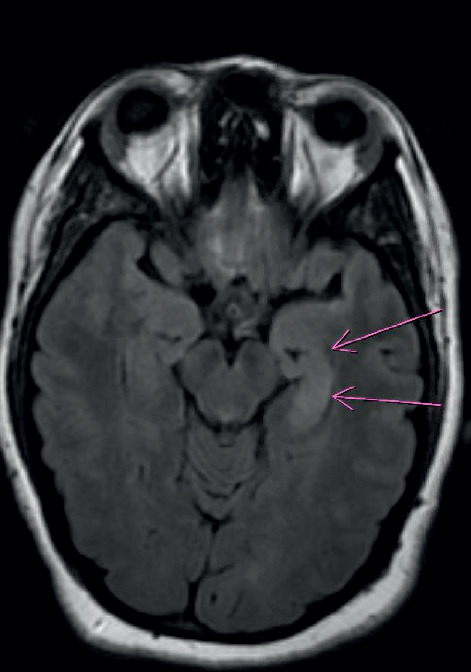
Brain MRI showing T2 flair hyperintensity in the medial temporal lobes with left lobe (pink arrows) revealing greater hyperintensity than right lobe.

**Figure 2:**
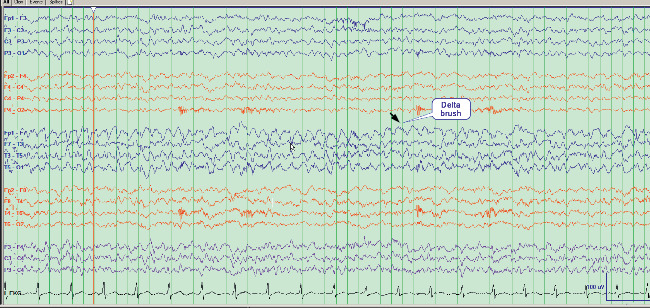
EEG showing electroclinical seizures exhibiting delta brushes.

Several days later, the patient was readmitted to a neighboring hospital for continued symptoms. A brain MRI revealed bilateral (left more than right) temporal lobe fluid-attenuated inversion recovery (FLAIR) hyperintensity (Figure 1). A test of the CSF revealed 53 white blood cells (WBC) (98% lymphocytes) and 2 oligoclonal bands. An electroencephalogram (EEG) revealed status epilepticus characterized by onset of discharges from the left frontocentral and left fronto-temporal region, accompanied by delta brushes (Figure 2). A test for anti-NMDAR antibodies showed presence in the serum and CSF (1 : 64), consistent with a diagnosis of NMDAR encephalitis. Given the inflammatory CSF, the patient was given 1000 mg of methylprednisolone IV and 0.4 gm/kg of IVIG for 5 days. Antiepileptic drugs (AED) started included levetiracetam, lacosamide, and lamotrigine. She was also maintained on risperidone. However, the patient remained with frequent seizures, behavioral agitation, and psychotic symptoms. CT of the chest, abdomen, and pelvis showed no evidence of neoplasm and a transvaginal ultrasound was negative for ovarian teratoma or other tumors. Given her recalcitrant course and presence of antibodies, on day 27 of status, she was started on 375 mg/m^2^ of rituximab weekly for 4 weeks. This resulted in clinical and electrographic improvement: normalized EEG and resolution of psychosis and agitation, with a return to baseline cognition and personality. Since then, she has remained on lacosamide 200 mg twice a day and rituximab 1000 mg IV every 6 months without recrudescence of symptoms.

### 2.2. Case 2

A 72-year-old female with a history of hypertension and anxiety presented after a motor vehicle collision with sudden loss of awareness while driving. The patient endorsed complete amnesia regarding the collision until her admission to the emergency department. The family also noted a recent three-month history of sporadic episodes of confusion lasting less than a minute in which the patient suddenly would become unaware of her surroundings, become pale with a blank stare, or display inappropriate behaviors such as getting up from her seat during dinner and spitting food into a vase, as well as a dystonic posturing of her right face and right upper limb concerning for faciobrachial seizures. She abruptly returned to baseline with no recollection of the event.

The patient continued to have seizures during the hospital course that was treated with levetiracetam. Initial EEG revealed background slowing suggesting mild encephalopathy, but no seizures were reported. The patient also exhibited cognitive decline with a Montreal Cognitive Assessment (MoCA) score of 18, with difficulty in areas of executive functioning, delayed recall, orientation, and abstraction. MRI revealed no acute process. She was discharged after a negative workup with recommendations for outpatient CSF studies and follow-up.

**Figure 3:**
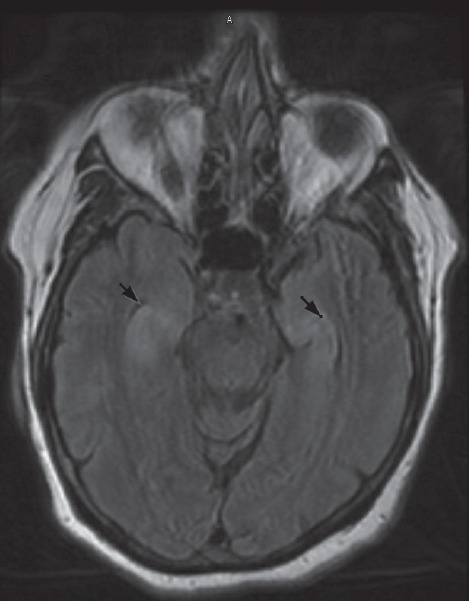
Brain MRI revealing symmetric signal abnormality, swelling of the hippocampus and right mesial temporal lobe, and a T2 FLAIR signal abnormality.

**Figure 4:**
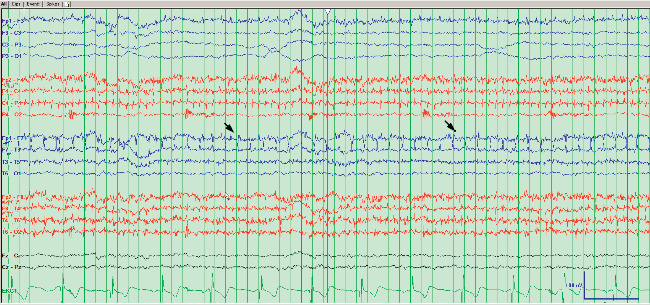
EEG showing background slowing and generalized slowing compatible with diffuse cortical dysfunction. EEG Also showing temporal lobe discharges.

She was readmitted to the hospital several weeks later for rapid cognitive decline, and increased frequency of staring episodes, up to 28 times per day. The patient's outpatient CSF workup results revealed an elevated white blood cell count, 4 oligoclonal bands, and elevated 14-3-3 levels. The CSF also eventually returned positive for anti-LGI1. Note that 14-3-3 is a nonspecific marker of cerebral damage and is not specific for Creutzfeldt–Jakob disease. Repeat brain MRI revealed FLAIR hyperintensity in the bilateral hippocampi, right mesial temporal lobe, and mild diffusion restriction in the right insular cortex without contrast enhancement or hemorrhage (Figure 3). On re-review of the first EEG, it was found to have left temporal discharges likely responsible for the seizures (Figure 4). A repeat EEG during this hospitalization was done and found to be abnormal, demonstrating a slower background than the prior EEG and overall having a generalized intermittent rhythmic slowing consistent with an encephalopathic picture. The left temporal originating epileptiform discharges and electrographic seizures were not seen on the repeat EEG.

Given the lack of response to AED and inflammatory-appearing CSF, autoimmune encephalitis was deemed to be the diagnosis. 1000 mg of methylprednisolone IV was started and continued for 5 days, resulting in improvements in the faciobrachial seizures and marked improvements in cognitive function to near-baseline. She was discharged on chronic prednisone that was tapered over 1 year. The CSF tested during her second hospital admission eventually returned positive for antibodies against LGI1.

Once the prednisone was discontinued, she had recrudescent cognitive decline and agitation requiring inpatient care. Repeat CSF revealed the presence of anti-LGI1 antibodies. She was treated acutely with 1000 mg methylprednisolone IV and 0.4 gm/kg IVIG for 5 days, which resulted in partial improvement. However, she had persistent expressive aphasia and impaired 5-minute recall. She was then given 1000 mg of rituximab IV resulting in an electrographic and clinical seizure freedom with return of premorbid cognitive function. The patient is currently maintained on 1000 mg of rituximab IV every 6 months.

### 2.3. Case 3

**Figure 5:**
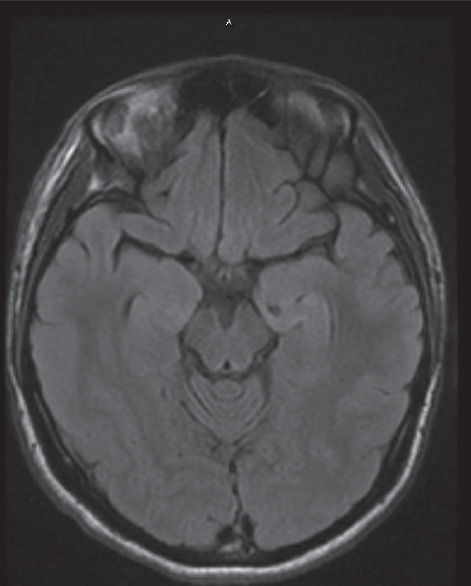
Normal brain MRI.

**Figure 6:**
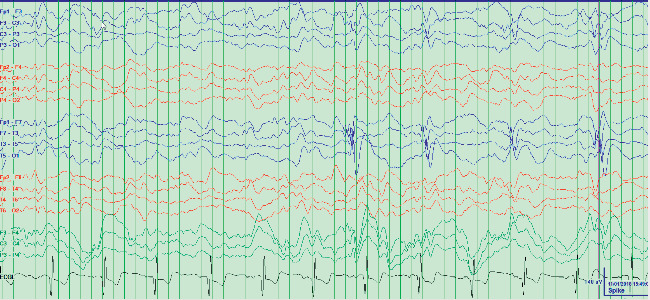
EEG showing left fronto-temporal sharp waves with intermittent slowing.

A 19-year-old male presented with high-grade fever, malaise, and complex partial seizures characterized by facial twitches, staring spells, and rigidity followed by brief unresponsiveness with amnesia. Over a few days, the syndrome progressed to status epilepticus recalcitrant to pharmacotherapy. His workup for infectious etiology was found to be negative. Initial MRI was unremarkable (Figure 5), and the CSF revealed 18 WBC (91% lymphocytes). His EEG showed left fronto-temporal sharp waves with intermittent slowing (Figure 6). Given the inflammatory CSF findings, he was treated with 0.4 gm/kg of IVIG and 1000 mg of methylprednisolone IV for 5 days with minimal clinical response and was subsequently administered 1000 mg of rituximab IV with abrogation of seizures and return to baseline functioning. Autoimmune encephalitis testing was negative in the CSF, but serum was found positive for N-type anti-VGCC antibodies, with titer levels at 0.28 (normal <0.02), obtained via send-out to Mayo Clinic Labs.

He has since demonstrated a full recovery and remains asymptomatic on 1000 mg of rituximab IV every 6 months. Antibodies to the voltage-gated N-type calcium channel are classically associated with Lambert–Eaton syndrome, a paraneoplastic neuromuscular disorder resulting from non-small-cell lung carcinoma. Our patient underwent a CT chest, and it was found to be negative.

## 3. Discussion

Intractable epilepsy remains a significant medical challenge, causing recurrent and prolonged hospitalizations and ICU stays. Autoimmune epilepsy is emerging as a treatable cause of intractable epilepsy [1]. It remains controversial whether the antibodies are directly pathogenic or markers of the disease. Common antigens include LGI1, CASPR2, AMPAR, GABAR, and NMDAR.

These antibodies are variably associated with cancer, in which case they are termed paraneoplastic. Autoimmune paraneoplastic syndromes are hypothesized to be due to cross-reactivity of antitumor antibodies binding to structurally similar but unrelated native neuronal antigens [2].

The incidence of autoimmune encephalitis has increased recently—from 0.4/100,000 to 1.2/100,00 person-years over the last 20 years—mostly due to increased awareness of autoimmune encephalitis and improved access to antibody-detection methodologies.

As illustrated by our series, the presentation of autoimmune encephalitis can mimic epilepsy due to other causes. Historical clues include a subacute decline in memory, cognition, seizures, and alterations of consciousness. Another hallmark is the presence of psychiatric or limbic symptoms such as psychosis, aggression, paranoia, or inappropriate sexual behavior in the absence of a psychiatric disorder. However, a diagnosis of autoimmune encephalitis cannot be made on the basis of semiology alone.

Each antibody correlates with specific clinical and electrographic features [3]. For instance, anti-NMDAR encephalitis is frequently associated with psychosis and delta brush on EEG [4]. Anti-LGI1 encephalitis is frequently associated with faciobrachial dystonic seizures, but the electrographic appearance can be variable depending upon the brain region involved (typically cortex, basal ganglia, and hippocampus) [5].

Delay in diagnosis and treatment has been associated with a worse prognosis and increased risk of relapse [6]. To reduce the risk of false-positive antibody results, we suggest the use of cell-based assays, as this methodology offers increased sensitivity and specificity compared to ELISA, RIA, or Western blot methods. In our patient with VGCC encephalitis, our titer of 0.28 is comparable to that of other reports of N-type VGCC encephalitis [6–9]. Therefore, this antibody is likely truly present and associated with our patient's syndrome. The question of pathogenicity may be raised, but this cannot be answered without mechanistic studies. The history of antibody-associated disease—whether SLE, NMO, MOG, or RA—is characterized by identification of antibody association years or decades prior to determining whether a given antibody is pathogenic and identifying the mechanism of pathogenesis. The focus of our report is purely clinical and we cannot make conclusions about the pathogenesis of any of the antibodies based upon our data. Ultimately, an accurate diagnosis requires the identification of an inflammatory syndrome and the correlation of any identified antibodies with its described clinical syndrome.

Typically, autoimmune encephalitis responds to immunomodulation or immunosuppression [5]. A single-center prospective study of 220 patients with anti-NMDAR encephalitis was done in which 99.5% received immunosuppressants including glucocorticoids, IVIG, and plasmapheresis (either individually or combined) and 7.3% received rituximab or cyclophosphamide (either individually or combined). In the first 12 months, 94.1% of patients improved, 2.3% died, and 17.3% had a relapse [10]. A case series of 5 patients with anti-LGI1 encephalitis showed that rituximab was efficacious and well tolerated as a treatment option [11]. Prior case studies of anti-VGCC associated with autoimmune encephalitis have shown that treatment with high-dose IV steroids combined with IVIG or plasmapheresis resulted in marked improvement of symptoms and resolution of seizures [12, 13]. Treatment response should be evaluated clinically since antibody titers do not correlate with disease activity [11].

All patients in this series met diagnostic criteria for autoimmune encephalitis and required immunosuppressive therapy for seizure control [12, 14–16]. Besides rituximab, options include IVIG, plasma exchange, chronic glucocorticoid therapy, azathioprine, mycophenolate mofetil, and cyclophosphamide. All three patients described are clinically normal on AED and rituximab only. None of our patients have developed serious infusion reactions, life-threatening infections, or cancer. Following treatment, behavioral disturbances and seizure activity ceased in all three patients and progression of the disease as well as permanent impairment of executive function was prevented.

## 4. Conclusion

We suggest that off-label use of rituximab may be an option in the acute and long-term management of intransigent autoimmune epilepsy. Early initiation of aggressive therapy can lead to normalization of clinical function. Randomized trials are required to establish the safety and efficacy of rituximab in this setting.

## Abbreviations

ICU: Intensive care unit
FLAIR: Fluid-attenuated inversion recovery
WBC: White blood cells
AED: Antiepileptic drugs
CSF: Cerebrospinal fluid
CT: Computed tomography
EEG: Electroencephalogram
MRI: Magnetic resonance imaging
LP: Lumbar puncture
NMDAR: *N*-methyl-D-aspartate receptor
IV: Intravenous
IVIG: Intravenous immunoglobulin
LGI1: Leucine-rich, glioma inactivated 1
CASPR2: Contactin-associated protein 2
VGCC: Voltage-gated calcium channel
AMPAR: *α*-Amino-3-hydroxy-5-methyl-4-isoxazolepropionic acid receptor
GABAR: Gamma aminobutyric acid receptor
MoCA: Montreal Cognitive Assessment.
